# Loss of *ZmLIPOXYGENASE4* Decreases *Fusarium verticillioides* Resistance in Maize Seedlings

**DOI:** 10.3390/genes12030335

**Published:** 2021-02-25

**Authors:** Alessandra Lanubile, Virginia Maria Grazia Borrelli, Mario Soccio, Paola Giorni, Lorenzo Stagnati, Matteo Busconi, Adriano Marocco

**Affiliations:** 1 Department of Sustainable Crop Production, Università Cattolica del Sacro Cuore, Via Emilia Parmense 84, 29122 Piacenza, Italy; virginiamaria.borrelli@unicatt.it (V.M.G.B.); paola.giorni@unicatt.it (P.G.); lorenzo.stagnati@unicatt.it (L.S.); matteo.busconi@unicatt.it (M.B.); adriano.marocco@unicatt.it (A.M.); 2 Research Center for Biodiversity and Ancient DNA, Università Cattolica del Sacro Cuore, Via Emilia Parmense 84, 29122 Piacenza, Italy; 3 Department of Agriculture, Food, Natural resources and Engineering (DAFNE), University of Foggia, Via Napoli 25, 71122 Foggia, Italy; mario.soccio@unifg.it

**Keywords:** lipoxygenase, gene expression, *Fusarium verticillioides*, *Zea mays*, pathogen resistance

## Abstract

*Fusarium verticillioides* is one of the most relevant fungal species in maize responsible for ear, stalk and seedling rot, as well as the fumonisin contamination of kernels. Plant lipoxygenases (LOX) synthesize oxylipins that play a crucial role in the regulation of defense mechanisms against pathogens and influence the outcome of pathogenesis. To better uncover the role of these signaling molecules in maize resistance against *F. verticillioides*, the functional characterization of the 9-LOX gene, *ZmLOX4*, was carried out in this study by employing mutants carrying Mu insertions in this gene (named as UFMu*lox4*). In this regard, the genotyping of five UFMu*lox4* identified the mutant UFMu10924 as the only one having an insertion in the coding region of the gene. The impact of *ZmLOX4* mutagenesis on kernel defense against *F. verticillioides* and fumonisin accumulation were investigated, resulting in an increased fungal susceptibility compared to the inbred lines W22 and Tzi18. Moreover, the expression of most of the genes involved in the LOX, jasmonic acid (JA) and green leaf volatiles (GLV) pathways, as well as LOX enzymatic activity, decreased or were unaffected by fungal inoculation in the mutant UFMu10924. These results confirm the strategic role of *ZmLOX4* in controlling defense against *F. verticillioides* and its influence on the expression of several LOX, JA and GLV genes.

## 1. Introduction

Globally, maize is the most widely grown cereal crop, with a world production of more than one billion tons from about 200 million ha [1]. However, the safety of maize as a food and feed component is under threat throughout the world. A large number of pathogens can attack and colonize developing maize ears and kernels, thus compromising the quantity and quality of grain production [2,3,4]. In recent years, the impact of climate change has exacerbated the occurrence of disease outbreaks as well as the increase of mycotoxin risks in maize [5,6]. Mycotoxin contamination is strongly influenced by environmental factors, and its presence varies greatly from year to year, even in small geographic areas, due to microclimatic variation [7].

*F. verticillioides* (Sacc.) Nirenberg (teleomorph *Gibberella moniliformis*) causes severe diseases of maize ears, stalks and seedlings, and is endemic in nearly all maize fields during harvest [8,9]. *F. verticillioides* infection is crucial for the resulting production of fumonisins and their effects on animal and human health [10,11]. Fumonisins B_1_, B_2_ and B_3_ represent the most abundant forms, and due to their carcinogenic properties, the European Union has regulated them, setting maximum tolerable levels in animal feed and human food [12,13,14].

The development of resistant germplasm is one of the major components of the strategy of fungal disease and fumonisin management, but the quantitative nature of these traits and the many environmental variations make the achievement of resistant materials very difficult. For this reason, complete resistance to *F. verticillioides* infection and fumonisin contamination has not yet been attained [15,16,17,18,19,20]. The identification of candidate genes involved in the host plant resistance mechanisms to this fungus by means of quantitative trait loci (QTL) mapping studies and transcriptomics analysis could provide new insights on the genetic basis of the maize-fungus interaction and hasten the production of resistant hybrids [16].

When maize is attacked by *F. verticillioides*, an intracellular signaling cascade is initiated, including the activation of hormones that in turn target transcription factor families, promoting the downstream switching on of defense responsive genes for pathogenesis-related proteins phenylpropanoid metabolism and lipoxygenases (LOX) [21,22,23,24,25,26]. Plant LOX enzymes synthesize several signaling molecules, named oxylipins, and depending on their regiospecificity, are classified in two major subfamilies, 9-LOX and 13-LOX [27]. In the 9-LOX pathway, polyunsaturated fatty acids (PUFA) are converted to hydroperoxy-octadecadienoic acid (HPOD), hydroperoxy-octadecatrienoic acid (HPOT) and 9S-keto octadecatrienoic acid (9-KOT), most of them involved in defense against pathogens. On the other hand, 13-LOX catalyzes the conversion of PUFA into 13-HPOT, subsequently metabolized to jasmonates, as jasmonic acid (JA) and its derivatives, as well as green leaf volatiles (GLV).

In maize, a total of 13 different *LOX* genes (*ZmLOXs*) with varying functions, localization, and regulation have been reported [28,29]. Six genes encode 13-lipoxygenases (*ZmLOX7*, *ZmLOX8*, *ZmLOX9*, *ZmLOX10*, *ZmLOX11* and *ZmLOX13*), the best-studied pathway, while the genes *ZmLOX1*, *ZmLOX2*, *ZmLOX3*, *ZmLOX4*, *ZmLOX5*, and *ZmLOX12* encode 9-lipoxygenases. The plastidial *ZmLOX6* represents a novel LOX subfamily, distinct from 9- and 13-LOX, able to metabolize fatty acid hydroperoxides derived from the 13-LOX pathway [30]. The role of each specific *ZmLOX* is still unknown. Various authors have attempted to clarify the involvement of oxylipins in host–pathogen interaction by deleting *LOX* genes in maize and pointed out their contribution to plant development and pest/pathogen resistance [31,32,33,34,35,36,37].

In this regard, in previous work by [37], the involvement of *ZmLOX4* for normal JA biosynthesis in wounded kernels inoculated with *F. verticillioides* was shown by employing two *lox4* mutants *lox4-7* and *lox4-10*, respectively. In this study, five novel maize mutants carrying transposon Mu insertions in distinct regions of the *ZmLOX4* gene (hereafter named as UFMu*lox4*), together with inbred lines W22 and Tzi18, were tested for resistance to *Fusarium* seedling rot (FSR) caused by *F. verticillioides*, using an inoculation assay in germinating kernels. This method allowed a better evaluation of infection progress at both kernel and seedling stages [38,39,40]. UFMu*lox4* mutants were analyzed for the presence of Mu insertions in the *ZmLOX4* gene along with its expression, and only the mutant carrying a mutation in the coding region of the gene and showing the absence of *ZmLOX4* transcript was further assayed for the content of fumonisins, the expression of several selected genes involved in the LOX, JA and GLV pathways, and LOX enzymatic activity. Collectively, the results from this study provided new insights about the role of the *ZmLOX4* gene, confirming not only its requirement for maize defense at the seedling stage against *F. verticillioides* but also supplying new information about how the altered expression of this gene could, in turn, reduce the transcriptional levels of several LOX and GLV genes.

## 2. Materials and Methods

### 2.1. Maize Inbred Lines and UFMuLox4 Mutants

The maize inbred lines W22 and Tzi18 belong to the “Goodman” maize association population [41,42] and were previously evaluated as moderately susceptible and resistant to *F. verticillioides* infection, respectively [38,39]. Seeds were obtained from the USDA-ARS-NCRPIS (Iowa State University, Regional Plant Introduction Station, Ames, Iowa, United States, 50011-1170). The UFMu*lox4* mutants (UFMu12283, UFMu06517, UFMu03075, UFMu10924 and UFMu03258) belong to the UniformMu population and are freely distributed by the Maize Cooperation Stock Center using online tools maintained at MaizeGDB.org. Mutator-transposable element insertional mutagenesis of UFMu*lox4* mutants was in the genetic background of the line W22. The UFMu*lox4* mutants were introgressed in W22 background with three backcrosses, and homozygous F_3_ mutant plants were used (BC_3_F_3_). Inbreds and mutants were maintained by sibbing at the Department of Sustainable Crop Production, Università Cattolica del Sacro Cuore, Piacenza, Italy.

### 2.2. PCR Confirmation and Genotyping of Mu Insertions

To confirm the presence of Mu insertions in the *ZmLOX4* gene, genomic DNA was extracted from individual plants and analyzed by PCR using gene-specific primers as described by [43]. At least two gene-specific primers are required for each insertion (one upstream and one downstream of the insertion site), and these need to be compatible with a Mu TIR-specific primer available on MaizeGDB [43]. The gene-specific primers were designed with Primer3 [44] for 22–24 base oligomers with a predicted Tm between 55 and 60 °C (Table S1). Gene-specific primers that flanked the insertion site were first PCR-tested in pairs with the wild-type DNA from W22 in order to confirm the capacity of the selected primers to amplify the expected wild-type fragment. Thereafter, the specific primers were paired with the MuTIR specific primer TIR6 (Table S1). PCR was completed with Taq DNA polymerase and buffers from Promega (Promega Corp., Madison, WI, USA). The two-step PCR thermocycling conditions were: 1.94 °C for 1 min; 2.94 °C for 25 s; 3. 62 °C for 30 s; 4.72 °C for 1 min; 5.8–10 cycles of 2 to 4; 6.94 °C for 25 s; 7.56 °C for 30 s; 8.72 °C for 1 min; 9.26 cycles of 6 to 8; 10.72 °C for 10 min. For sequence validation of insertion sites, PCR reaction with TIR6 was carried out, PCR product was gel purified and sequenced using the TIR6 primer and/or gene-specific primer (Figure S1).

### 2.3. Inoculation Bioassay

The rolled towel assay (RTA) phenotyping method was used to artificially infect mature kernels of each inbred line (W22 and Tzi18) and the five UFMu*lox4* mutants [38,39,45,46]. Briefly, twenty seeds of a similar size and without visible damage were selected, 10 to be used as inoculated samples (*F. verticillioides* inoculation) and 10 to be used as control (mock inoculation). Six RTAs in total for mock (M) and inoculated (I) conditions were carried out for each inbred line and mutant. The seeds were surface sterilized as previously described in [38,39,45,46] and placed on two moistened towels of germinating paper (Anchor Paper, Saint Paul, MN, USA). The kernels were inoculated with 100 μL of 1 × 10^6^ conidial suspension of *F. verticillioides* ITEM10027 (MPVP 294). The fungal cultures were maintained on Petri plates (9 cm diameter) in potato dextrose agar (infusion from potatoes, 200 g; dextrose, 15 g; agar, 20 g; H_2_O to 1 L) and incubated at 25 °C with a 12 h photoperiod for 14 days. After the inoculation step, the towels were rolled up and maintained for 7 days at 25 °C in the dark. Controls were prepared as above but replacing the inoculation step with water. For each seedling, *Fusarium* seedling rot (FSR) severity, seedling length (SL) and fresh seedling weight (SW) of inbred lines and UFMu*lox4* mutants in mock (M) and inoculated (I) conditions were calculated. The FSR was assessed on each seedling by a visual evaluation of the seedling size and visible colonization of *F. verticillioides* using a scale from 1 to 5, as previously reported [38,39,45,46]. On this scale, 1 corresponds to the complete absence of disease symptoms, and five corresponds to the entire rotting of the kernel. For further biological analysis, kernels at 7 days post-inoculation (dpi) were collected and immediately frozen in liquid nitrogen and stored at −80 °C.

### 2.4. Analysis of Total Fumonisin Content

Five g of sample ground in liquid nitrogen with a pestle and mortar were weighed and placed in an extraction tube. Twenty-five mL of AQUA buffer (VICAM, Watertown, MA, USA) was measured with a graduated cylinder and poured into the extraction tube. The extraction tube was vortexed for 2 min at maximum speed. The extract was then filtered into a clean extraction tube. For the quantification of total fumonisins, 100 μL of filtered extract were transferred to the Fumo-V AQUA strip test (VICAM) by dropping (~1 drop/second) vertically into the circular opening. After allowing the strip test to develop for 5 min on a flat surface, the Fumo-V strip test was inserted into the Vertu reader, and the results were read. The values of fumonisins (B_1_ + B_2_ + B_3_) given in the text are expressed in mg/kg.

### 2.5. RNA Extraction and Real-Time RT–PCR Expression Analysis

Total RNA extraction and purification were performed according to [47,48]. Real-time reverse transcription-PCR (RT–PCR) experiments were performed on kernels collected at 7 dpi using the 2× iQ SYBR Green Supermix (Bio-Rad, Hercules, CA, USA) and the CFX-96 device (Bio-Rad, Hercules, CA, USA). One µg of total RNA was used for cDNA synthesis using the iScript cDNA synthesis kit protocol (Bio-Rad, Hercules, CA, USA). Twenty ng of single-strand cDNA determined by fluorometric assay (Qubit, Invitrogen, Carlsbad, CA, USA) were used for real-time RT–PCR. The relative RT–PCR conditions applied were: 95 °C for 3 min and 40 cycles at 95 °C 15 s, 57–63 °C for 30 s, followed by a melting curve analysis [47,48]. Three technical replicates (within each biological replicate) were employed for each tested sample and template-free negative controls. Gene-specific primers are listed in Table S2. The reference housekeeping gene *β-actin* was used to normalize the expression of the target genes, and FC values in gene expression were calculated using the 2^−ΔΔCt^ method [49].

### 2.6. Determination of Lipoxygenase Enzymatic Activity

The maize kernels were ground using a Retsch mixer mill with two 30 s cycles at 15 Hz. For each sample, 1 g of the ground powder was resuspended in 4 mL of 100 mM Na-phosphate buffer (pH 7.0) and placed in an ice-water bath, stirred for 1 h and then centrifuged twice at 25,000× *g* at 4 °C for 20 min. The obtained supernatant was stored in an ice-water bath and used daily to measure lipoxygenase activity. Protein content was determined according to the method of Lowry, as modified by [50], using bovine serum albumin as a standard. Total lipoxygenase activity was evaluated spectrophotometrically by means of a PerkinElmer λ 45 UV-vis spectrometer (PerkinElmer, Wellesley, MA, USA) according to [51] by monitoring the absorbance increase at 234 nm due to the conversion of linoleate into the corresponding hydroperoxide. The assay medium was 2 mL of 100 mM Na-acetate buffer (pH 5.5) containing 400 µM Na-linoleate and 1 µL Tween-20 per µmol linoleate. The reaction was started by adding 0.05–0.10 mg of protein extract. One unit of lipoxygenase corresponded to the formation of 1 µmol of conjugated diene per min.

### 2.7. Statistical Analysis

For analysis of the RTA phenotypic values, standard deviations of the means were calculated on 10 seedlings of three RTAs. For fumonisin content, gene expression and enzymatic activity analysis, standard deviations of the means were calculated on three biological replicates. One-factor ANOVA, followed by Tukey’s HSD test (*p* < 0.05), was performed on the phenotypic values, fumonisin content, FC values of all genes and lipoxygenase enzyme activity to set significant differences among genotypes. For RTA phenotyping analysis, differences between mock and inoculated RTAs within the same line were performed using two-factor ANOVA and considered to be significant at * *p* ≤ 0.05; *** *p* ≤  0.001.

## 3. Results

### 3.1. Molecular Analysis for Transposon Mutagenesis in the ZmLOX4 Gene

**Figure 1:**
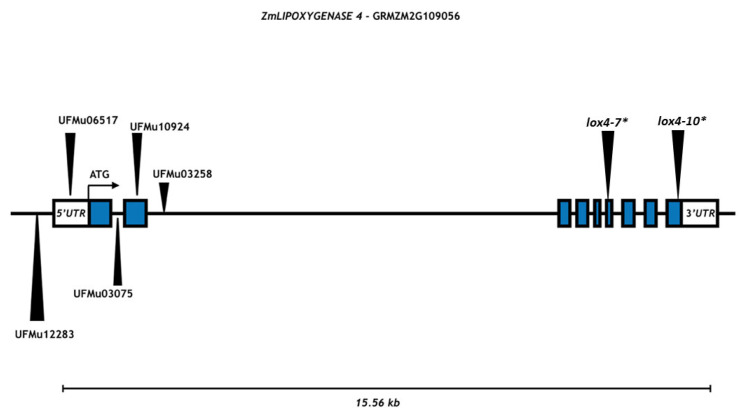
Schematic representation of the genomic structure of the *ZmLIPOXYGENASE4* gene showing the mutator element insertion sites. Blue boxes and black lines represent exons and introns, respectively. * indicates the mutator element insertional sites for *lox4-7* and *lox4-10* mutants as previously reported in [37].

Mu transposon insertions in the *ZmLOX4* gene were confirmed by PCR amplification, followed by DNA sequencing (Figure 1, Figure S1). In a previous study by [37], two *lox4* mutants, *lox4-7* and *lox4-10*, had Mu insertion in exons 6 and 9, respectively (Figure 1). In this study, five novel UFMu*lox4* mutants were considered and the analysis of Mu element presence identified UFMu12283 as having an insertion at −204 bp from the ATG start codon, UFMu06517 at −52 bp from ATG in the 5′ UTR region, UFMu03075 and UFMu03258 at +717 and +1131 bp in introns 1 and 2, respectively, whereas UFMu10924 harbored a Mu element in exon 2 at +935 bp (Figure 1, Figure S1). The Mu transposon insertion in the coding region of the *ZmLOX4* gene observed for the mutant UFMu10924 could lead to a higher probability of obtaining a truncated protein during translation. For this reason, even though further disease phenotyping analysis and the expression of the *ZmLOX4* gene were carried out for all UFMu*lox4* mutants, the extensive molecular characterization and the enzymatic assay were performed on UFMu10924 only.

### 3.2. Phenotypic Analysis of Maize Inbred Lines and UFMulox4 Mutants

**Figure 2:**
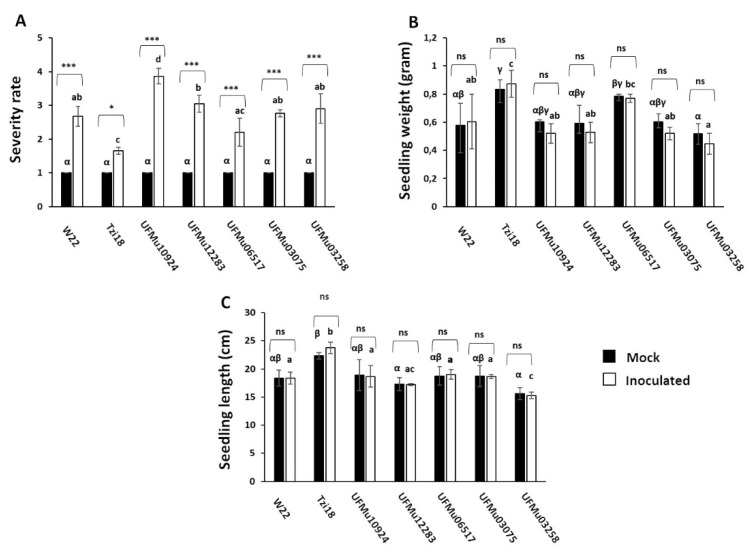
Phenotypic values of *Fusarium* seedling rot ((**A**), severity rate), seedling weight ((**B**), gram) and seedling length ((**C**), cm) in mock (black boxes) and inoculated (white boxes) RTAs for the tested maize inbred lines and UFMu*lox4* mutants. Asterisk (*) indicate significant differences between mock and inoculated RTAs within the same line, according to two-way ANOVA (* *p* < 0.05; *** *p* < 0.001). ns: not significant. Different Greek letters over the black histograms indicate significant differences among the mock RTAs of the tested lines, whereas different Latin letters over the white histograms indicate significant differences among the inoculated RTAs of the same lines, as resulting from Tukey’s honestly significant difference test (*p* < 0.05).

To determine whether *ZmLOX4* contributes to the resistance of maize to mycotoxigenic fungi, the levels of infection caused by *F. verticillioides* were tested in the five UFMu*lox4* mutants and in W22 and Tzi18 inbred lines (Figure 2). Different types of response to FSR were found through the RTA screening. The FSR severity rates ranged from 1.65 ± 0.10 to 3.86 ± 0.23, with Tzi18 the most resistant line and UFMu10924 the most susceptible. The inbred line W22 showed FSR values of moderate susceptibility with severity scores not significantly different from those observed for the remaining UFMu*lox4* mutants (Figure 2A). Conversely, the occurrence of *Fusarium* seedling rot in mock RTA was null (1 = healthy), with seedlings having no visible sign of fungal colonization for all lines (Figure 2A).

Morphologically, no significant differences were reported in seedling weight (SW) and length (SL) among W22 and the Mu insertional mutants, considering both mock and inoculated RTAs (Figure 2B,C). Interestingly, Tzi18 displayed the highest values of SW, before and after treatment, and SL after fungal treatment (*p* < 0.05). These findings confirm the better performances of the inbred line in terms of disease resistance and seedling growth [38,39].

Total fumonisin (B_1_ + B_2_ + B_3_) content was evaluated in inoculated RTAs 7 days after inoculation for UFMu10924, Tzi18 and W22 lines. No significant differences in fumonisin production were measured between UFMu10924 and W22, with total fumonisin concentrations of 58.94 ± 10.48 and 55.46 ± 3.78 mg/kg, respectively, whereas fumonisin production was significantly (*p* < 0.05) reduced almost 11-fold on Tzi18 kernels (5.10 ± 1.44 mg/kg). These results collectively demonstrated the resistance of the latter line, as highlighted by the reduced FSR values and the low fumonisin content.

**Figure 3:**
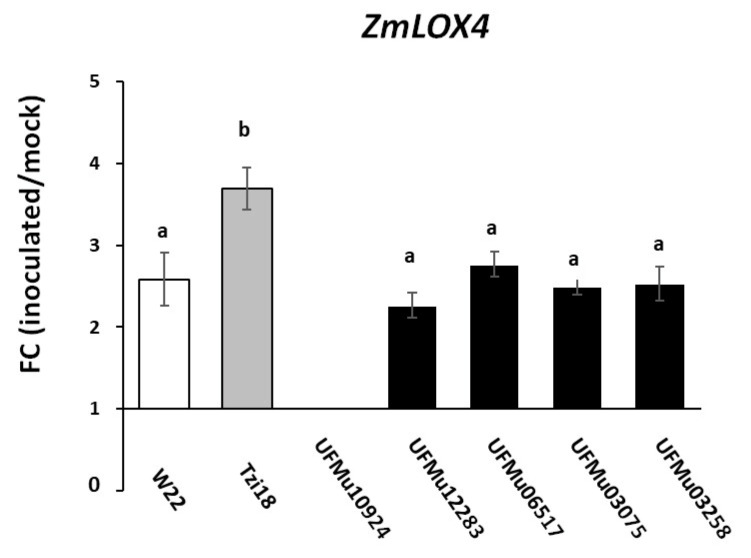
Real-time RT–PCR analysis of *ZmLOX4* gene. Fold change (FC) of expression between inoculated and mock kernels of W22, Tzi18 and UFMu*lox4* lines at 7 dpi with *F. verticillioides*. Vertical bars indicate ± sd. Different letters over the histograms indicate significant differences among means of the three lines, resulting from Tukey’s honestly significant difference test (*p* < 0.05).

**Figure 4:**
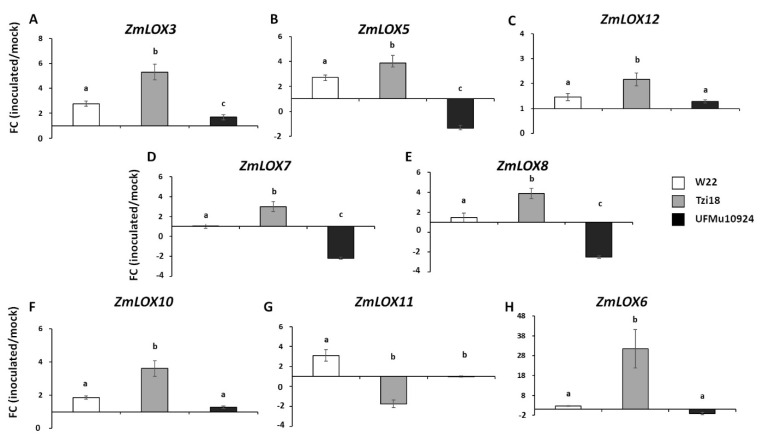
Real-time RT-PCR analysis of *ZmLOX* genes. Fold change (FC) of expression between inoculated and mock kernels of W22, Tzi18 and UFMu10924 lines at 7 dpi with *F. verticillioides* of (**A**) *ZmLOX3*, (**B**) *ZmLOX5*, (**C**) *ZmLOX12*, (**D**) *ZmLOX7*, (**E**) *ZmLOX8*, (**F**) *ZmLOX10*, (**G**) *ZmLOX11* and (**H**) *ZmLOX6*. Vertical bars indicate ± sd. Different letters over the histograms indicate significant differences among means of the three lines, as resulting from Tukey’s honestly significant difference test (*p* < 0.05).

The expression levels of the *ZmLOX4* gene were first assayed by real-time RT–PCR in all five UFMu*lox4* mutants, Tzi18 and W22 lines at 7 dpi with RTA bioassay (Figure 3). The expression profiles were reported as fold change (FC) of inoculated over-mock inoculated kernels. As expected, no discernable transcript was detected in the mutant UFMu10924, whereas the transcripts of *ZmLOX4* were strongly expressed in Tzi18. Lower values of FC were detected in W22 that did not result significantly different from the other four UFMu*lox4* mutants (Figure 3). Besides the *ZmLOX4* gene, the transcriptional changes of three selected 9-LOX genes, *ZmLOX3*, *ZmLOX5* and *ZmLOX12*, four 13-LOX genes, *ZmLOX7*, *ZmLOX8*, *ZmLOX10* and *ZmLOX11*, and the plastidial *ZmLOX6* were analyzed considering only Tzi18, W22 and the UFMu10924 mutant (Figure 4).

The three lines showed differences in the expression of almost all genes. The upregulation of most of them occurred in the resistant line Tzi18, excluding *ZmLOX11*, which was slightly downregulated with a FC value of −1.75 (Figure 4G). The most highly induced gene was *ZmLOX6,* with an increase of up to 31.55-fold (Figure 4H), followed by *ZmLOX3* with the induction of up to 5.30-fold (Figure 4A). Slightly lower FC values were reported for the other assayed genes in the same line, with FCs ranging from 3.88 to 2.17 (Figure 4). Overexpression was also detected for *ZmLOX* genes in the W22 line, albeit at more moderate levels, varying from 3.10 for *ZmLOX11* to 1.10 for *ZmLOX7* (Figure 4G,D, respectively).

In contrast to Tzi18 and W22, the expression of most *ZmLOX* genes decreased or was unaffected by *F. verticillioides* inoculation in the mutant UFMu10924 (Figure 4). Significant differences with both inbred lines were observed for the genes *ZmLOX3*, *ZmLOX5*, *ZmLOX7* and *ZmLOX8*, where the lowest levels of expression were reached with the latter gene (FC = −2.52; Figure 4E).

**Figure 5:**
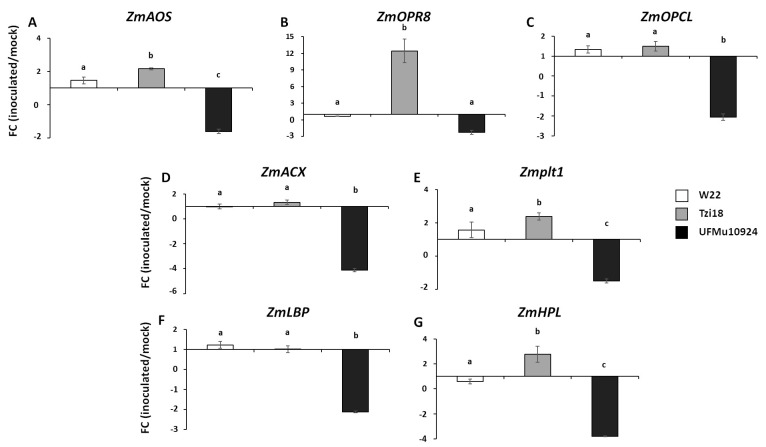
Real-time RT-PCR analysis of JA and GLV genes. Fold change (FC) of expression between inoculated and mock kernels of W22, Tzi18 and UFMu10924 lines at 7 dpi with *F. verticillioides* of (**A**) *ZmAOS*, (**B**) *ZmOPR8*, (**C**) *ZmOPCL*, (**D**) *ZmACX*, (**E**) *Zmplt1*, (**F**) *ZmLBP* and (**G**) *ZmHPL*. Vertical bars indicate ± sd. Different letters over the histograms indicate significant differences among means of the three lines, as resulting from Tukey’s honestly significant difference test (*p* < 0.05).

Regarding the genes involved in the synthesis of jasmonates and green leaf volatiles encoding for allene oxide synthase (*ZmAOS*), 12-oxo-phytodienoic acid (12-OPDA) reductase (*ZmOPR8*), OPC-8:0 CoA ligase 1 (*ZmOPCL*), acyl-CoA oxidase (*ZmACX*) and hydroperoxide lyase (*ZmHPL*), as well as those encoding for lipid transfer proteins, more precisely, phospholipase transfer protein homolog 1 (*Zmplt1*) and lipid-binding protein (*ZmLBP*), similar expression patterns were reported, even if a less marked reaction was observed as compared to *ZmLOX* genes (Figure 5).

The resistant line Tzi18 showed the strongest induction for all genes contrasting with W22 and UFMu10924. The most relevant difference was found for the gene *ZmOPR8*, for which *F. verticillioides* treatment triggered a response about 12 times higher in the resistant line than both the other lines (Figure 5B).

In contrast with the gene induction observed for the Tzi18 line, the expression of JA and GLV genes appeared to be not notably affected by the fungal inoculation in W22, whereas the levels of transcripts of all genes were decreased in mutant UFMu10924, with FC ranging from −4.12 to −1.51 (Figure 5).

**Figure 6:**
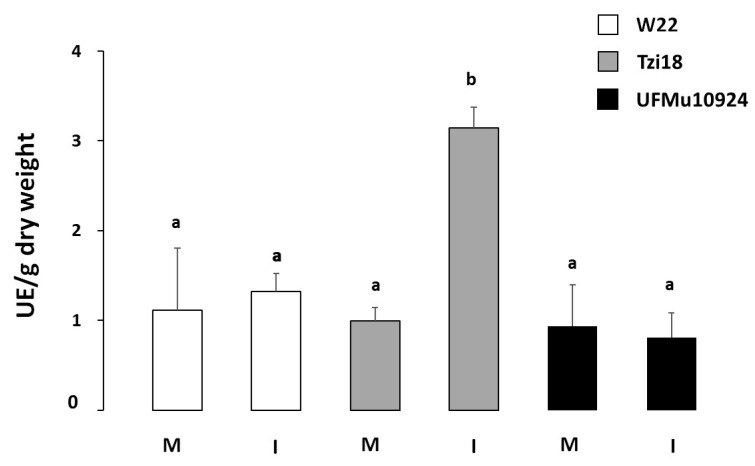
Total enzymatic lipoxygenase (LOX) activity expressed as UE/g of dry weight in kernels of W22, Tzi18 and UFMu10924 lines before (M, mock) and 7 dpi with *F. verticillioides* (I). Vertical bars indicate ± sd. Different letters over the histograms indicate significant differences among means of the three lines, resulting from Tukey’s honestly significant difference test (*p* < 0.05).

Given the diversified transcriptional changes induced by *F. verticillioides* observed in Tzi18, W22 and UFMu10924 lines, the study was extended to lipoxygenase enzymatic activity. The assays were conducted on the same two inbred lines and the UFMu*lox4* mutant by comparing mock and inoculated kernels, and total enzymatic LOX activity was expressed as UE/g of dry weight (Figure 6).

In mock kernels, LOX results were not significantly different for all three lines (UE/g of dry weight = 1.11, 0.99 and 0.93 for W22, Tzi18 and UFMu10924, respectively). On the other hand, a significant increase of the LOX activity (*p* < 0.05) was elicited by fungal treatment in the Tzi18 resistant inbred line (UE/g of dry weight = 3.14). Albeit no significant, a mild higher LOX activity was reported for W22, whereas no change occurred in the mutant UFMu10924.

The lower LOX activity found in the W22 line and, more markedly, in the UFMu*lox4* mutant probably reflects the reduced FC values previously described for the LOX and GLV genes, and once again, it points out the relevance of the *ZmLOX4* gene in the resistance mechanisms towards *F. verticillioides*.

## 4. Discussion

Plant oxylipins, including GLV and hormone JA, play a key role in signaling molecules to induce defense responses against pathogens [27]. To better elucidate the role of oxylipin-mediated crosstalk in the plant-fungal interaction of maize germinating kernels and *F. verticillioides*, a functional analysis was performed with maize mutants carrying mutator insertions in the *ZmLOX4* gene in comparison with the wild-type inbred line W22 and the resistant genotype Tzi18.

In this study, the analysis of five novel Mu transposon insertions identified mutant UFMu10924 as the only one having an insertion in the W22 *LOX4* gene coding region. Analysis of *ZmLOX4* gene expression showed the absence of transcript in this mutant, whereas W22, Tzi18 and the remaining UFMu*lox4* mutants exhibited a higher induction after *F. verticillioides* inoculation. These results revealed that transposon insertion in exon 2 observed for UFMu10924 was the only one to cause lack of expression. Similar findings were previously reported in [52], where *ZmLOX4* transcripts were strongly accumulated in wild-type plants and not for exonic insertion events in the *lox4-7* and *lox4-10* mutants, considered as null alleles of *ZmLOX4*.

The effects of transposon mutagenesis on kernel defense against *F. verticillioides* were further investigated to prompt the hypothesis of *ZmLOX4* contribution to pathogen resistance. In this regard, the RTA screening method was employed to evaluate *F. verticillioides* establishment and growth in germinating maize kernels. This phenotyping bioassay has the capacity to score multiple uniformly grown seedlings for each tested genotype. The seedlings scored in the RTA grew simultaneously, in controlled conditions, and inoculated with a common stock of fungal conidia, avoiding possible external injury or stress effects. The UFMu10924 mutant was found to be the most susceptible to fungal infection compared to W22 and the other knockout mutants having the insertions in the non-coding regions of the *ZmLOX4* gene, whereas Tzi18 was confirmed to be the most resistant line with the lowest FSR values. These findings were in line with a previous study, where *F. verticillioides* colonized more aggressively and produced a higher number of conidia on *lox4* and *lox5* mutant kernels compared to wild-type kernels [37]. Moreover, without a functional *ZmLOX4* gene, seeds did not accumulate the JA hormone precursor, 12-OPDA, and JA itself at normal levels, suggesting that 9-oxylipin products from LOX4 are required for JA biosynthesis in *F. verticillioides* infected seeds [37]. Previous research investigated two independent Mu transposon insertions in the 9-lipoxygenase *LOX4*, UFMu01831 and UFMu03303, and reported a higher *Spodoptera exigua* caterpillar mass compared to wild-type W22, indicating that not only maize defense mechanisms to fungal infection were affected by *ZmLOX4* Mu insertions, but also a defense to insect herbivory [53].

Because *F. verticillioides* represents the main pathogen producing fumonisins and mycotoxin contamination constitutes a significant agroeconomic threat, it was of concern to assay whether disruption of this gene had any effect on fumonisin production in the inoculated kernels. RTAs showed that UFMu10924 kernels were highly contaminated by fumonisins (B_1_ + B_2_ + B_3_), slightly more than the W22 line, albeit these differences were not significant. On the other hand, the significantly reduced levels of fumonisin contamination determined in Tzi18 demonstrated the superiority of this inbred line and its possible employment as a source of resistance. A low relationship between disease severity and production of fumonisins, as observed for UFMu*lox4* mutant and W22, could be an inherent factor of the pathosystem maize-*F. verticillioides*, hypothesis reinforced by several studies. It was suggested that genetic factors that affect grain infection might act independently of those affecting fumonisin production, and the quantitative genetic nature of these two traits could partly explain the presence of mycotoxins even in case of the absence of infection [54,55,56,57].

Regarding gene expression profile, the 9-LOX genes (*ZmLOX3*, *ZmLOX5* and *ZmLOX12*), the 13-LOX genes (*ZmLOX7*, *ZmLOX8* and *ZmLOX10*) and *ZmLOX6* were always upregulated in the resistant Tzi18 line, followed a more moderate increment in W22, while *lox4* mutant did not show any difference of gene expression or even a downregulation. Previous experiments reported an earlier and enhanced induction of *LOX* genes in maize kernels belonging to the CO441 resistant line at 3 and 7 dpi with *F. verticillioides* compared to the CO354 susceptible line [48].

A different trend was observed for the *lox3* mutants as described in the works by [32,37]. In contrast to wild-type kernels, earlier and significantly higher levels of expression were observed for the *ZmLOX4*, *ZmLOX5* and *ZmLOX12* genes in *lox3* mutant kernels in response to *F. verticillioides*. Moreover, *lox3* mutants exhibited a reduced fungal colonization and fumonisin production, leading to the conclusion that *ZmLOX3* can be considered a susceptibility gene in this pathosystem [32,37]. In this study, the lack of expression of *ZmLOX4* in the UFMu10924 mutant caused downregulation of all *ZmLOX* genes, including *ZmLOX3*. The absence of transcriptional changes observed for this gene should have increased resistance against *F. verticillioides*, as reported in previous studies [32,37]. Conversely, a different behavior was described in this work. It could be speculated that the effect of the impaired *ZmLOX3* gene expression and the possible consequent reduced levels of LOX3-derived oxylipins, as the linolenic acid-derived 9-HOD(T)E, were invalidated by the concerted underexpression of the other seven *ZmLOX* genes, more specifically *ZmLOX5* and *ZmLOX12*. The function of these two genes, together with *ZmLOX4*, was already demonstrated to be fundamental for the normal production of jasmonate, the major hormone involved in the plant defense mechanisms against *F. verticillioides* [36]. This hypothesis will need to be better supported through the analysis of JA hormone content and 9-LOX oxylipin profile, as well as the employment of double *lox3lox4* mutants, not currently available, could better clarify the contrasting roles of these two genes.

The expression profile of the 13-LOX *ZmLOX11* differed from the other *ZmLOX* genes, showing downregulation of almost 2-fold in the resistant Tzi18 line, contrasting to that in W22, whereas the UFMu*lox4* mutant did not accumulate any transcript for this gene. *ZmLOX11* was hypothesized to be involved in osmotic stress response, and it has been observed to be induced in maize leaves by abscisic acid [58]. Previous RNA-sequencing experiments found that *ZmLOX11* was not significantly differentially expressed in maize interactions with toxigenic and atoxigenic strains of *Aspergillus flavus,* comparing resistant and susceptible inbred lines [29]. In addition, Dolezal et al. [59] reported a downregulation in the maize kernels of B73 at 4 dpi with the same fungus, suggesting a more marginal implication of this gene in kernel defense against pathogens.

This study also included the evaluation of genes for the production of jasmonates and GLV. JA biosynthesis starts from the AOS branch of the 13-LOX pathway with the conversion of (9Z, 11E, 15Z)-(13S)-hydroperoxyoctadeca-9,11,15-trienoate (=13S-HPOTE) to allene oxide by AOS, the formation of 12-OPDA by an allene oxide cyclase, the further production of oxophytoenoic acid by an OPDA reductase (OPR), followed by three reactions of β-oxidation [28,60]. The main genes implicated in JA biosynthesis, *ZmAOS*, *ZmOPR8*, *ZmOPCL* and *ZmACX*, were all downregulated in the UFMu10924 mutant; in contrast, the resistant line Tzi18 exhibited the highest FC values, and this was particularly pronounced for the gene *ZmOPR8*. Similar observations were reported for the *lox12* mutants, displaying a reduced expression of the JA-regulating gene *ZmLOX10* and the JA biosynthesis gene *ZmOPR8* [36]. Furthermore, the analysis of double mutants *opr7 opr8* showed a lack of JA production, strong developmental defects and severe disease symptoms to insects and pathogens, including *F. verticillioides* [34]. Additionally, a notable decrease of *ZmOPR7* and *ZmOPR8* gene expression was also ascertained in *lox10* mutants, where the diminished levels of JA and compromised resistance to insect feeding demonstrated the central role of this hormone in resistance mechanisms against fungal and insect attack [35,36].

In a previous study, a different trend was described for the *lox3* mutants that accumulated *ZmOPR8* transcripts more abundantly in roots infected with root-knot nematode *Meloidogyne incognita* compared to the wild-type roots [33]. Furthermore, elevated levels of expression were reported for the *ZmHPL* gene in both mock and infected *lox3* mutant roots [33]. In maize, GLV production is determined by 13-LOX LOX10 activity in conjunction with HPL, responsible for C18:2 or C18:3-derived 13-hydroperoxy fatty acids cleavage into C6 aldehydes and C12-oxo-fatty acids [28,61]. In the present work, the *ZmHPL* gene was downregulated in UFMu10924 to a greater extent than W22, while it was induced in the resistant Tzi18 line. It is tempting to speculate that the reduced transcript levels of *HPL* in the *lox4* mutant could, in turn, impair the expression of genes involved in JA production. The connections between GLV and JA biosynthesis pathways after *F. verticillioides* infection need to be further determined to better understand the role of GLV in the regulation of JA production.

Lipid metabolism has multiple roles in defense against pathogens, plant cell physiology, growth and development [62]. LTPs belong to the most functionally important classes of plant proteins binding and transferring lipids. In this study, the expression analysis of two LTPs, *Zmplt1* and *ZmLBP*, was investigated, and as for LOX, JA and GLV genes, a reduction of FC values was described for the *lox4* mutant compared to W22, whereas a higher induction was observed in the Tzi18 only for the *Zmplt1* gene. Both genes were differentially regulated in kernels of resistant and susceptible maize lines at seven dpi with *F. verticillioides* [48]. Furthermore, the expression of a nonspecific lipid transfer protein (ns-LTP-1) was enhanced in a resistant wheat line at 72 h post-*F. graminearum* infection [63].

Earlier investigations demonstrated that levels of *LOX* transcripts paralleled LOX activity in several host–pathogen combinations. Hwang and Hwang (2010) reported that infection with *Xanthomonas campestris* pv *vesicatoria* stimulated a more elevated expression of the *CaLOX1* gene and a parallel more induced LOX activity during incompatible interactions in pepper leaves [64]. Similarly, *LOX* transcript accumulation and LOX enzymatic levels were coincident in potato-resistant plants after *Phytophthora infestans* treatment [65].

In this study, greater levels of LOX enzymatic activity were measured in the inoculated kernels of Tzi18. This trend was in line with the enhanced resistance to *F. verticillioides*, the lower fumonisin accumulation and the decreased LOX, JA and GLV gene expression values described for this genotype. In contrast, during compatible interactions between *lox4* mutant/W22 lines and the fungus, no significant enzyme accumulation was observed, and this pattern could reflect the lower gene expression. Although the differences in *ZmLOX* transcript accumulation were more marked between the UFMu10924 and wild-type W22, no significant changes were found at an enzymatic level between the mutant and the inbred line. A possible explanation for the missing correlation between gene expression and enzyme activity could be due to the fact that only the measurement of total LOX activity, determined by several *LOX* genes and not the different LOX enzyme isoforms, was considered in this study. Moreover, the UFMu10924 mutant is in the same genetic background as the wild-type W22 that even did not show any induction of total LOX enzyme activity. Further experiments, including later times of infection and oxylipin analysis, will clarify these findings more accurately. In addition, backcrossing between the UFMu10924 mutant and the resistant line Tzi18 will allow to better evaluate the effect of the mutation in the *ZmLOX4* gene in a resistant background.

In conclusion, the knowledge gained from the characterization of the *ZmLOX4* gene in maize defense against *F. verticillioides* and its influence on genes of the LOX, JA and GLV pathways, together with additional investigations, as hormonal and lipid profiles, will allow the employment of *LOX* genes and resistant genotypes, as Tzi18, to hamper fungal infection in maize via marker-assisted selection and genome editing approaches.

## Data Availability

All data generated or analyzed during this study are available from the corresponding author on reasonable request.

## Supplementary Materials

The following are available online at https://www.mdpi.com/2073-4425/12/3/335/s1, Table S1: Primer sequences for UFMu*lox4* mutants genotyping, Table S2: Primer sequences for real-time RT–PCR analyses, Figure S1: UFMu*lox4* sequences for transposon insertion confirmation.
