# Supplementary material for: Loss of ZmLIPOXYGENASE4 Decreases Fusarium verticillioides Resistance in Maize Seedlings
Source: Genes (Basel). 2021 Feb 25;12(3):335. doi: 10.3390/genes12030335 (PMC7996282; doi:10.3390/genes12030335)
Supplement: Supplementary file 1 [file genes-12-00335-s001.pdf]

*Article*

# Loss of *ZmLIPOXYGENASE4* Decreases *Fusarium verticillioides* Resistance in Maize Seedlings

**Alessandra Lanubile [1,2]*, Virginia Maria Grazia Borrelli [1], Mario Soccio [3], Paola Giorni [1], Lorenzo Stagnati [1], Matteo Busconi [1,2] and Adriano Marocco [1,2]**

[1] Department of Sustainable Crop Production, Università Cattolica del Sacro Cuore, Via Emilia Parmense 84, 29122 Piacenza, Italy; virginiamaria.borrelli@unicatt.it (V.M.G.B.); paola.giorni@unicatt.it (P.G.); lorenzo.stagnati@unicatt.it (L.S.); matteo.busconi@unicatt.it (M.B.); adriano.marocco@unicatt.it (A.M.)

[2] Research Center for Biodiversity and Ancient DNA, Università Cattolica del Sacro Cuore, Via Emilia Parmense 84, 29122 Piacenza, Italy

[3] Department of Agriculture, Food, Natural resources and Engineering (DAFNE), University of Foggia, Via Napoli 25, 71122 Foggia, Italy; mario.soccio@unifg.it (M.S.)

* Correspondence: alessandra.lanubile@unicatt.it (A.L.), Tel.: (+39-0523-599206)

**Table 1.** Primer sequences for UFMu*lox4* mutants genotyping.

| UFMu*lox4* mutant | Primer pairs for ZmLOX4 WT allele | Primer pairs for UFMu insertion |
|---|---|---|
| **UFMu12283** | fD CAGTCATCATCGACTCATCGTT + rM ACAACTGTTCCATTCCATGTCC | TIR6 AGAGAAGCCAACGCCAWCGCCTCYATTTCGTC + fD CAGTCATCATCGACTCATCGTT |
| **UFMu06517** | fD CAGTCATCATCGACTCATCGTT + rM ACAACTGTTCCATTCCATGTCC | TIR6 AGAGAAGCCAACGCCAWCGCCTCYATTTCGTC + fD CAGTCATCATCGACTCATCGTT |
| **UFMu03075** | fI CCTATGGAAGATTGGAAGTCGA + rO GCCATCTCAATCGCATTGAATG | TIR6 AGAGAAGCCAACGCCAWCGCCTCYATTTCGTC + rO GCCATCTCAATCGCATTGAATG |
| **UFMu10924** | fL GTTTTGTGTGGTAGGGCTTGCGTT + rT TCCTGAGTAAGAATACTCACGTCG | TIR6 AGAGAAGCCAACGCCAWCGCCTCYATTTCGTC + rT TCCTGAGTAAGAATACTCACGTCG |
| **UFMu03258** | fC CTCTCAGTCATCATCGACTCAT + rT TCCTGAGTAAGAATACTCACGTCG | TIR6 AGAGAAGCCAACGCCAWCGCCTCYATTTCGTC + fC CTCTCAGTCATCATCGACTCAT |

**UFMu12283: primers: TIR6 + fD; sequenced with fD; sequence code FR07686683; transposon in blue.**

GTACCTCTTTTTTTTCACCTTTTCGTATACACGTCGCTGTCGCCTCCATCGCTGGGCTGGCAAGAGAACGCGAGGC
GAAGCAGCCGCGGCCGCCCGCCTATTTATCGCGAGATAATTGCCATTATGGACGAAGAGAGGAGGTGATTCGA
CGAAATGGAGGCGTTGGCGTTGGCTTCTCACAGATGTTAAGGTTGTCAACTCGAGGGGGGCACCCTGGACCACA
CCTGGGCATCCACTCGTACGCCGTTTAGATTTTCTTCTTACAAAGGACCACTCACGGGCTTTTTCCAACCCCCAG
CTATGGCTACGCTTCCCTTGTTGATGATATATTGCCC

**UFMu06517: primers: TIR6 + fD; sequenced with fD; sequence code FR07686686; transposon in blue.**

GTCCGTCTTTTTTTTCACCTTTTCGTATACACGTCGCTGTCGCCTCCATCGCTGGGCTGGCAAGAGAACGCGAGGC
GAAGCAGCCGCGGCCGCCCGCCTATTTATCGCGGGCTCGCGGCACAGGGCAGCGGCAGTTCCATACATACATA
CACCAACCGTGCGTGAAGGAAGGCCTTTGCTCGCCGCCACATCACATTGGCAGGCGAGGCGAGGGAGCGAGCA
GCAGGGCAAGGCATCCACACCCACACCCACGAGATAATTGCCATTATAGACGAAGAGCGGACGGGATTCGAC
GAAATGGAGGCGTTGGCTGAGCTTCTCTTGACG

**UFMu03075: primers: TIR6 + rO; sequenced with rO; sequence code FR07686692; transposon in blue.**

GGTAACTTACTTTTTTTTCACCTTTTCGTATACACGTCGCTGTCGCCTCCATCGCTGGGCTGGCAAGAGAACGCGA
GGCGAAGCAGCCGCGGCCGCCCGCCTATTTATCGCGAGATAATTGCCATTATGGACGAAGAGAGGAGGTGATT
CGACGAAATGGAGGCGTTGGCGTTGGCTTCTCACAGGCGGAGCTGCTGGTTAATCATACCTTTAAGTTGTCTGCA
AAAAGATGTCAGCGGAAGCGGCACCGCACAGGGCGGTTTGTGGTTGGTTCCCATTGATACCTTGCATAGTGCTA
GCTTCTTGATTAGATCCACGTAAAGCACACTGATGATCAAAGTCACCTACGTTAACTTCCCATTAGCCCACACCT
CCTCCTCCAACGACTTATACATAAGCGTGCAATATTGAACTCCAAAAGACGGTAGATTGGAGTAATCCAAATTT
AGAAATGAACTTGTCAGCGGGTCCTCCACTCGTGGACCCAGGTTGTTGGAAGTACACGTGGAGAGTGTACATTG
CATATTGTGATTCCCCCAGAACACTTCAGTGTGACATCACTCTCTCCATAAAATATACTAGCTATCGATCCCCAT
CTGTAGATTAGTCACTGTGTAAGCTGACTCCGGACACCAGACATACTTCCCTGATACTGTCGAGTAGACACGTTG
CTCTTGGTAGACCGGCACATAGGCTAGATGCGATGACGCAGACGATTCGCAGGGGTGATGAGCGCGCAGCGCA
TCTACCTCCTGAAGTTTGAGTTATGTTTTATTTTTCTAACTGTCGGTAGATCGAATAAGAGCTAGCAGCTCAGAGA
ATACTAGTTGACGA

**UFMu10924: primers: TIR6 + rT; sequenced with rT; sequence code FR07686682; transposon in blue.**

CGAGTACAAAAGACCGAGGGGAGAATACTCACGTCGTTGGCGAGAAGACGCGTTCCTGGGAGTAGAGATTGTG
CGGGTAGATCCACGAGTTGGCGACGAAGACGACGGTGCCCCTGCCGGGGACGCCCTCGAGGGTGAGCGACTTG
AGGAAGAACTCGGCGTGCTGCAGGTTCCTGACCAGGACGGCGCCCGGGACGCCCTGCGACCCGTCCCACTCGA
AGCTCACCCGGTACAGAGATAATTGCCATTATAGACGAAGAGCGGACGGGATTCGACGAAATGGAGGCGATGG
CGCGGCTTCTCAGTGCGTCCAGTTGAGAGAGAACATTATCAAGAAAAATGAGACAAGGCGGCTTAAATCTTATT
CTAGGCGGGAGGTGTTATTATCCCCGAGGCGGCACACAGCACAAAAGCTTGTAAGTTACTGTGCACCTCTCGGG
TTAAAAGTTATGTGAAAAGTTTCATGTTTTAGGAACATTCTTCTTCTCTTCTGCTTAACATGTTGCTCCTTTTTTTTC
TTGCTCTTCTTCTTCGTCTTCCCAACTTGTTCTTCCGTTTCTTATTTGAAGACAAAAAAAACCGGAGTAAGAAGTG
CTGACTTCTCTTTCATGACTTTCTGACCACCTAATCATAGGTAATGTAGTAGCTACTGTTGCTTTTCTTCACTCCTC
CCCCATTATCATATTTTCGGTTTCTTCTATAACCAATTGTTCCTTTCTGAACCTTGTAGGATGTGAATTGTTGCAGC

CAAAAAACTTGTTGATTGAGTCTGGAAACGTCTCACTGATGAGGCCGCCCTTGCTTTTTGAGTCTTCCACCACCT
AGATAAAACACACACTTGATGGATGGGCAAGCAGTAACTTGCGT

**UFMu03258: primers: TIR6 + fC; sequenced with fC; sequence code FR07686690; transposon in blue.**

GACTTCTTTTTCATTTCTGTCCGGGGTTCGGGTTCCCAGGTTCCCAGTTCACACTTGAGGGGTGGGCCCTATCGCC
CTCTCCATTCCATGCGGCCGGCTGTTTTTTTTCGAGCAGTTTGCCCCACCACTCTTACCTGTAACATGTTCCTTATT
ACACCGGAGGGCTGTCGCTTGAACCCTTTGAGGCATGCTCTCCTGGTTAACAATTGCTTTGGTTGGTATGCCTTTG
TGCATGTCAACGGACACTTTCACCTCACACATGCCTTTTAGACTGGATGGGATTGTAGACCACGGATACGTCAG
AATCGTTGAATGATTTATGGGAAGCACTCCGAACATCTTACCGTCACCATCATGATAAGAAATCCCCTGACTTTT
TCCATAAACGACTTATAAATCCTCGTGCCATATTGCACTATATAAGACCTTTCGTGGGAGTCAGACAATCTTGGG
TAAGAACTTTAATGTGGTGGAAAACTACTCACCGCACGCTTTTAGAAATTAAGATCAAGACTGTACAGGGCATA
TGGAGATTTTTAAAACTATTTGAGAGTGATACTCATTATACCCCACTCGTAGAATTGCTGCTACGTGATCTTTAGG
ATCAGCCGATGGACGAACACAGAAGAATCCCCCCAACATAATCTCGCTGAGCATGTAGGCCGAGTCTTCCCCC
CTCCACACCCCAGGCAGTTTCGACACATAAGAGGTAGCTAAACAAGAAAATCGCATGTAGGTAGACATCAACT
GTGAGAGTCATGCTTGCGATTGATGAGTAGTGATCATCGCCAGCAATCAAACCTGACCGGCAAGTACCCCCGCA
TCCTAGGAGCCCAGATTGAATACTAATA

**Figure 1.** UFMu*lox4* sequences for transposon insertion confirmation.

**Table 2.** Primer sequences for real-time RT-PCR analyses.

| Gene | Primer forward | Primer reverse | Source |
|---|---|---|---|
| ***ZmLOX3*** | CGTTCCTCATCGACGTCAAC | GCGTGTAGACCTTGCTCTTG | AF329371.1 Maschietto et al. 2015 |
| ***ZmLOX4*** | TACCGTGACACGCTGAACAT | CACAGCCACACCTCTCTTGA | DQ335762.1 Maschietto et al. 2015 |
| ***ZmLOX5*** | CCGAGTTCTTCCTCAAGACG | CCTGGGAGTAGAGCTTGTGC | DQ335763.1 Maschietto et al. 2015 |
| ***ZmLOX6*** | GTGTACACTCCACCGGACAG | GTTGAGCCAGTGAGTGACGA | DQ335764.1 Maschietto et al. 2015 |
| ***ZmLOX7*** | GCAATCATGCCAGGCGAGGCCGAGGG | GAGCACGGCGACCGCGCCCGGCTC | GRMZM2G070092 This paper |
| ***ZmLOX8*** | AGATCCAGGAGAACAGCGAG | GATCATGGACGGAGGAGGAG | DQ335766.1 Maschietto et al. 2015 |
| ***ZmLOX10*** | GACGAGTGCAACAACAACCT | TGCATGCTGAGGATGGATCA | DQ335768.1 Maschietto et al. 2015 |
| ***ZmLOX11*** | TCGTGGTTCAAAGACGAGGA | ATCTCCTTTGTGATGGCGGA | DQ335769.1 Maschietto et al. 2015 |
| ***ZmLOX12*** | AACAAGGGGTGTGCGTCTAC | TCATTGACGGAGACATGAGC | DQ335770.1 Maschietto et al. 2015 |
| ***ZmAOS*** | GATGAGCAACGACTTCACGA | CACACGCACACACACAAAAA | NM_001111774.1 Maschietto et al. 2015 |
| ***ZmOPR8*** | TACTGATGCCCGATGGATCC | AACCTGCTTTGATGGCGTTT | AY921645.1 Maschietto et al. 2015 |
| ***ZmOPCL*** | GTGCCCATGTTCCACGTCTA | AGCATGGCGACGAGGATG | NM_001148670.1 Maschietto et al. 2015 |
| ***ZmACX*** | GTCCTCGTCTTCCACGTTGT | CGAGGTCAAGACCAAAGCTC | GRMZM2G864319 Maschietto et al. 2015 |
| ***Zmplt1*** | ATATCTATCCCGCCGTCGTC | TCCGTCTCCTCTCTCTCTCA | NM_001111841.1 Maschietto et al. 2015 |
| ***ZmLBP*** | TTCGACACATCAAGCTTTGG | ACGCAAGCCATATCAGCTCT | GRMZM2G155555 Maschietto et al. 2015 |
| ***ZmHPL*** | ATCTTCCGGTTCCTCTGCAA | AAGGAGTGGATGAGCAGCTC | AY540745.1 Maschietto et al. 2015 |
| ***Zmβ-actin*** | ATGGTCAAGGCCGGTTTCG | TCAGGATGCCTCTCTTGGCC | AY273142.1 Maschietto et al. 2015 |
